# Exploring *Bemisia tabaci* Middle East–Asia Minor I and Mediterranean Cryptic Species Relationship with Cowpea Mild Mottle Virus and Their Dynamics in Soybean Fields

**DOI:** 10.3390/insects15080624

**Published:** 2024-08-19

**Authors:** Felipe Barreto da Silva, Rodrigo de Sarandy Raposo, Sarah Forlani de Campos, Juliana Uzan, Julio Massaharu Marubayashi, Marcos Roberto Ribeiro-Junior, Angélica Maria Nogueira, Caroline da Cruz Martines, Vinicius Henrique Bello, Cristiane Müller, Maria Márcia Pereira Sartori, Renate Krause-Sakate

**Affiliations:** 1 School of Agricultural Sciences, São Paulo State University (UNESP), Botucatu 18610-034, Brazil; rodrigo.raposo@unesp.br (R.d.S.R.); sarah.campos@unesp.br (S.F.d.C.); j.uzan@unesp.br (J.U.); juliommagro@hotmail.com (J.M.M.); marcosrrjr@gmail.com (M.R.R.-J.); axnogueira@hotmail.com (A.M.N.); caroline.martines@unesp.br (C.d.C.M.); vhbello@hotmail.com (V.H.B.); maria.mp.sartori@unesp.br (M.M.P.S.); 2 Gulf Coast Research and Education Center, University of Florida, Wimauma, FL 33598, USA; 3 Corteva™ Agrisciences, Mogi Mirim 13814-000, Brazil; cristiane.muller@corteva.com

**Keywords:** whiteflies, insect vectors, virus–vectors interaction, transmission efficiency, whitefly survey, soybean disease

## Abstract

**Simple Summary:**

Cowpea mild mottle virus (CPMMV) transmitted by the invasive whitefly *Bemisia tabaci* is a concern for soybean production in Brazil. The Middle East–Asia Minor 1 (MEAM1) and Mediterranean (MED) cryptic species efficiently transmit the virus and resist conventional insecticides, presenting significant challenges to control. Our results indicate a statistically significant difference in the acquisition, inoculation, and retention of CPMMV by *Bemisia tabaci* MEAM1 and *B. tabaci* MED cryptic species, with MED being significantly more efficient at transmitting CPMMV compared to MEAM1. *Bemisia tabaci* MED can also transmit the virus with a shorter acquisition period compared to that of MEAM1, being considered a better vector of CPMMV. Additionally, over three soybean seasons (2019/2020, 2020/2021, and 2021/2022), MEAM1 was predominant across most sites visited, but MED co-occurred with MEAM1 in different places, showing the advancement of this pest in open field areas of Brazil.

**Abstract:**

Cowpea mild mottle virus (CPMMV, genus *Carlavirus*, family *Betaflexividae*) is an economically important virus infecting soybeans in Brazil, where it was initially identified in 1983. CPMMV is transmitted by the whitefly, *Bemisia tabaci*, and occasionally by seeds. Over the last three decades, the most invasive *B. tabaci* Middle East–Asia Minor 1 (MEAM1), and lately the Mediterranean (MED) cryptic species, have replaced the indigenous species in Brazil, with MEAM1 being predominant. In this study, we investigated the transmission properties of CPMMV by MEAM1 and MED, and their distribution in major soybean-growing areas in São Paulo State. Our results from transmission assays with a single insect revealed that MED is a more efficient vector compared to MEAM1, transmitting the virus within a two-minute inoculation access period. *B. tabaci* MEAM1 is still the predominant whitefly species in São Paulo State, but MED was also identified in different places, mainly in mixed infestations with MEAM1. Some areas transitioned to a predominance of MED over the three years, while others, where MED had previously been detected, showed a reduction in the insects during the same period. Understanding the transmission dynamics of CPMMV and the distribution of its vectors is crucial for implementing effective management strategies to control the virus spread and protect soybean crops. Further research into the mechanisms driving the shifts in whitefly species dominance and CPMMV distribution will be essential for sustaining soybean production in Brazil.

## 1. Introduction

Cowpea mild mottle virus (CPMMV), a member of the family *Betaflexividae* and the genus *Carlavirus*, was initially identified in Brazil, infecting common beans in 1983 [1], and later soybeans in the 2000/2001 season [2], causing stem necrosis [3]. The symptoms of stem necrosis are no longer observed in the soybean genotypes currently used in Brazil, which express symptoms of mosaic patterns, chlorosis, vein clearing, mottling, and leaf deformation [4,5,6]. Diverse symptoms in the same plant or the occurrence of symptomless infections can also occur [5,6]. The losses in soybean production can vary from 174 to 638 kg ha^−1^ depending on the genotype used and the age of the plant at infection [6].

CPMMV is transmitted by the polyphagous whitefly *B. tabaci* (Gennadius) (Hemiptera: Aleyrodidae), and according to the literature, it spreads in a non-persistent manner via the B biotype [7,8,9]. *Bemisia tabaci* poses a major threat to various cultivated plants and is recognized as one of the world’s 100 most invasive pests [10]. This pest is also listed by the Ministry of Agriculture, Livestock, and Supply (MAPA) as one of the most important invader pests in Brazil (https://www.gov.br/agricultura/pt-br/assuntos/sustentabilidade/tecnologia-agropecuaria/recursos-geneticos-1/especies-introduzidas) accessed on 1 December 2023. Molecular phylogenic analysis based on the mitochondrial cytochrome oxidase I (mtCOI) gene indicates that *B. tabaci* is a species complex consisting of at least 44 morphologically indistinguishable species [11,12,13]. The species within the complex also differ in characteristics, such as the host plant range, the capacity to cause plant disorders, attraction by natural enemies, endosymbionts, the expression of resistance, and plant virus transmission capabilities [14,15,16,17,18].

*Bemisa tabaci* Middle East–Asia Minor 1 (MEAM1), formerly known as the ‘B biotype’ and *Bemisia argentifolii* (Bellows & Perring [19]) is a globally distributed species. Another widely distributed species is *B. tabaci* Mediterranean (MED), formerly known as the ‘Q biotype’ and considered as *B. tabaci* sensu stricto [20,21]. Both MEAM1 and MED are recognized as the most invasive cryptic species within the *B. tabaci* complex, presenting significant challenges to control [22,23]. In Brazil, MEAM1, which was introduced in the early 1990s [24], is predominant [25]. On the other hand, MED was initially reported in the southernmost region of Brazil in 2014, infesting sweet pepper crops [26], and has since spread across areas of the country particularly associated with ornamental plants and vegetable crops [25,27,28,29]. Recently, MED has also been identified as colonizing soybean plants in open-field conditions [30]. This discovery raises concerns for soybean production, given the lower susceptibility of MED to some conventional insecticides [31,32].

Soybean, one of the world’s most important sources of animal feed and vegetable oil, stands out as the most economical crop in Brazil, the leading producer worldwide [33]. In the 2023 summer season, Brazilian production reached 156.00 million metric tons, accounting for 39% of global soybean production, surpassing the United States [34]. In São Paulo State, soybean is gaining prominence as an important agricultural crop. In 2021, the total cultivated area in São Paulo State reached 1.29 million hectares [35]. Whiteflies are considered one of the most important pests for this crop due to direct damage that occurs through phloem feeding, indirect damage leading to sooty mold fungi (*Capnodium* sp.) production that interferes with photosynthesis [36], and through the transmission of viruses [37].

To understand the dynamics of MEAM1 and MED in soybean fields, we surveyed whiteflies in the most representative soybean-producing areas of São Paulo State. Transmission assays were conducted to evaluate the acquisition, inoculation, and retention periods, comparing the transmission efficiencies of CPMMV by both the cryptic species in soybean plants. As MED has recently started to spread and colonize soybeans under field conditions, while MEAM1 is well established, understanding their relationship with CPMMV is essential for developing better integrated whitefly and virus management strategies.

## 2. Material and Methods

### 2.1. Transmission Properties of CPMMV by Single B. tabaci MEAM1 and MED Insects

#### 2.1.1. Insects

A population of MEAM1 (GenBank access: MF624473) was collected from cabbage (*Brassica oleracea*) in Campinas, São Paulo, Brazil, and maintained on cabbage plants. The MED population (GenBank access: KX673609) was initially collected from bell pepper (*Capsicum annuum*) in Óleo, São Paulo, Brazil, and maintained on cotton plants. For whitefly identification, total DNA acid extraction was carried out following a modified Chelex protocol [38]. DNA was used as a template for PCR analysis to differentiate MEAM1 and MED using the Bem23 primer pair: Bem23F and Bem23R [39]. The primer sequences and annealing temperatures of PCR reactions used for whitefly identification are available in Supplementary Table S1. This primer pair amplifies a microsatellite locus of about 200 bp and 400 bp for MEAM1 and MED, respectively [40]. Amplified DNA was visualized by electrophoresis in 2% agarose gel stained with ethidium bromide.

The whiteflies from each species used in the transmission experiments were newly emerged (48 h after emergence) and starved for 2 h before being used for all inoculation assays.

#### 2.1.2. Plants

Healthy and CPMMV-infected soybean cv. TMG 7062 IPRO were utilized. All the plants were maintained in insect-proof rearing cages (45 × 45 × 55 cm) until they reached their first trifoliate leaves (Vegetative Stage 1). Healthy and diseased plants were molecularly tested for CPMMV presence as described in Section 2.2. before each assay.

#### 2.1.3. Acquisition Access Period (AAP)

Approximately 300 whiteflies were released onto cages containing CPMMV-infected soybean plants. After acquisition access periods (AAPs) of 5, 10, 15, 20, and 30 min, and 1, 3, 6, and 24 h, the adults were collected randomly from the CPMMV-infected soybean leaves. Subsequently, a single adult was transferred into a clip cage placed on a healthy soybean plant. An inoculation access period (IAP) of 24 h was assessed for each plant. Following the IAP, all the whiteflies were removed, and the plants were kept in insect-proof cages. Thirty replicated plants were used for each AAP. Thirty days after inoculation, each plant was tested for CPMMV infection via molecular analysis as described in Section 2.2.

#### 2.1.4. Inoculation Access Period (IAP)

The whitefly adults were placed in cages with the CPMMV-infected plants. After 24 h of AAP, a single adult was collected and transferred to a clip cage attached to a healthy soybean plant with the first true leaf. IAPs of 2, 5, 10, 20, and 30 min, and 1, 2, 3, 6, 12, and 24 h were assessed. For each IAP, thirty replicated plants were used. After each IAP was assessed, all the whiteflies were removed, and the plants were kept in insect-proof cages. Thirty days after the IAP, each plant was tested for CPMMV infection via molecular analysis as described in Section 2.2.

#### 2.1.5. Retention Period (RP)

Cotton plants (*Gossypium hirsitum* L.), which are a host for MEAM1 and MED, but not for CPMMV, were the plants used to evaluate the retention of the virus in a non-host plant. Firstly, the whitefly adults were placed in cages with the CPMMV-infected plants, and after 24 h of AAP, the viruliferous MEAM1 and MED whiteflies were collected and transferred to a clip cage attached to cotton plants. After 5, 10, 15, 20, and 30 min, and 1, 3, 6, and 24 h on the cotton plants, the single whiteflies were collected and transferred to a clip cage attached to soybean plants. After 24 h, all the whiteflies were removed from soybean plants, and the plants were kept in insect-proof cages. Thirty replicated plants were used for each RP. Thirty days after the IAP, each plant was tested for CPMMV infection via molecular analysis as described in Section 2.2.

### 2.2. CPMMV RNA Extraction and Identification

Total RNA was extracted from each plant sample following the method described by Bertheau et al. (1998). Subsequently, a one-step reverse transcription-polymerase chain reaction (RT-PCR) was performed using AMV reverse transcriptase (Promega, São Paulo, Brazil) with the specific primer pair CPMMV1280-F and CPMMV1696-R, amplifying a region of the coat protein [37]. The primer sequences and annealing temperatures of PCR reactions used for CPMMV identification are available in Supplementary Table S1. The amplification protocol consisted of an initial step of 42 °C for 30 min, followed by 94 °C for 2 min, 30 cycles of 94 °C for 54 s, annealing at 54 °C for 50 s, and elongation at 72 °C for 50 s, followed by a final extension step at 72 °C for 10 min. The amplified DNA was visualized through electrophoresis in 2% agarose gel stained with ethidium bromide.

### 2.3. Distribution of Whitefly Species on Major Soybean-Growing Areas in São Paulo State

Whitefly Sampling

Field surveys were conducted during the growing summer seasons 2019/2020, 2020/2021, and 2021/202 in the major soybean-growing areas across São Paulo State. Adult specimens of whiteflies were collected from the underside of leaves randomly selected across soybean fields using a hand-held aspirator. These specimens were immediately transferred to tubes containing absolute ethanol and stored at −20 °C until the further molecular identification of whitefly species. Molecular analysis was performed as described in Section 2.1. For each population, 10 adults were analyzed and identified. 

### 2.4. Data Analysis

Transmission efficiency was defined as the ratio between the number of infected plants and the total number of whitefly-inoculated plants. The acquisition, inoculation, and retention comparisons of CPMMV by *B. tabaci* MEAM1 and MED were analyzed using the non-parametric Mann–Whitney test with a significance level of 0.01. Analyses were performed using the software R 3.6.1. [41].

## 3. Results

### 3.1. Transmission of CPMMV by MEAM1 and MED on Soybean Using Single Insects

**Figure 1:**
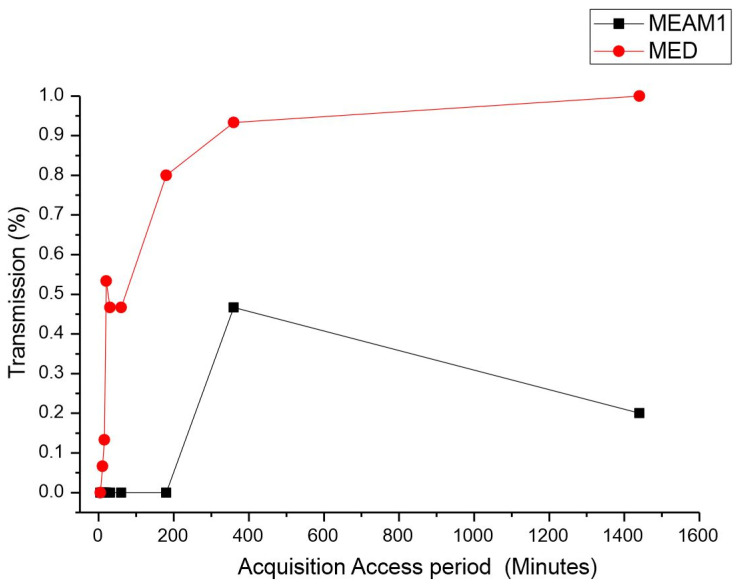
Effect of acquisition access period (AAP) on the transmission of cowpea mild mottle virus by *Bemisia tabaci* Middle East–Asia Minor 1 and *Bemisia tabaci* Mediterranean. The differences were estimated by the Mann–Whitney test at a significant level of 0.01 (*n* = 30).

*Acquisition access period (AAP).* The average time for the acquisition of CPMMV by the MEAM1 and MED adults is listed in Table 1. There was a significant difference between the acquisition access periods by the MEAM1 and MED species. The differences were estimated by the Mann–Whitney test at a significance level of 0.05 (*p*-value < 0.00001, adjusted for ties), as indicated by the percentage of CPMMV-infected plants exposed to a single virulent whitefly (Figure 1). For the short AAPs of 5, 10, and 15 min, the relative quantity of infected plants did not significantly differ between MEAM1 and MED. MEAM1 did not transmit CPMMV for the AAPs ranging from 5 min to 3 h. MED was able to transmit CPMMV for an AAP of 10 min. The CPMMV acquisition assessed for MED demonstrated that the higher the AAP was, the more efficient transmission was, reaching 100% efficiency at the maximum AAP evaluated, which was 24 h. MEAM1 was able to transmit CPMMV only for an AAP of 6 h, reaching the peak of transmission, and then it decreased for a 24 h AAP. Overall, MED was more effective in transmitting CPMMV, particularly for the longer AAPs.

**Figure 2:**
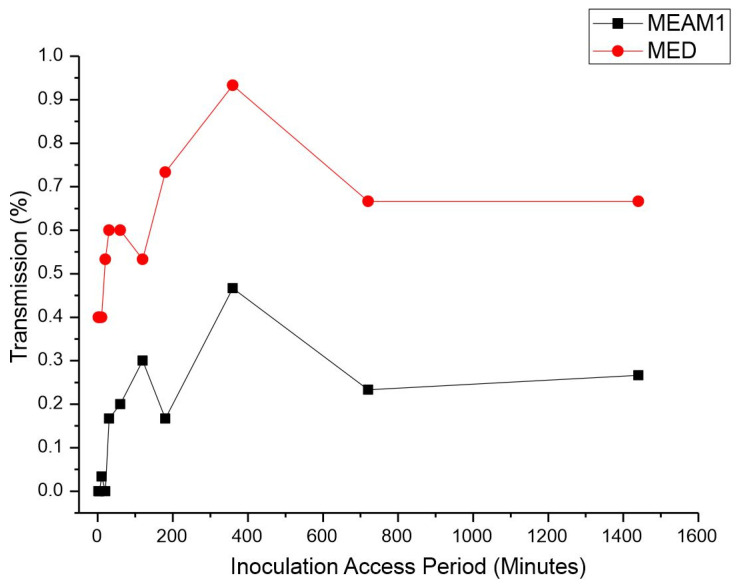
Effect of inoculation access period (IAP) on the transmission of cowpea mild mottle virus by *Bemisia tabaci* Middle East–Asia Minor 1 and *Bemisia tabaci* Mediterranean. The differences were estimated by the Mann–Whitney test at a significant level of 0.01 (*n* = 30).

*Inoculation access period (IAP).* The IAP was significantly different between the MEAM1 and MED adults according to the Mann–Whitney test *p*-value < 0.00001 at a significance level of 0.05 (adjusted for ties). The average retention period (RP) of CPMMV by the MEAM1 and MED adults is listed in Table 2. Single viruliferous MED was able to efficiently transmit CPMMV in the minimum assessed IAP. The efficient transmission of 40% by MED occurred within just 2 min of the IAP (Figure 2). The highest transmission rate by MED, 93.3%, was observed with an IAP of 6 h. Coincidentally, MEAM1 also reached its peak transmission rate of 46.6% with an IAP of 6 h. Differently from MED, MEAM1 was able to transmit significantly at a low rate of 30% after 2 h of the IAP.

**Figure 3:**
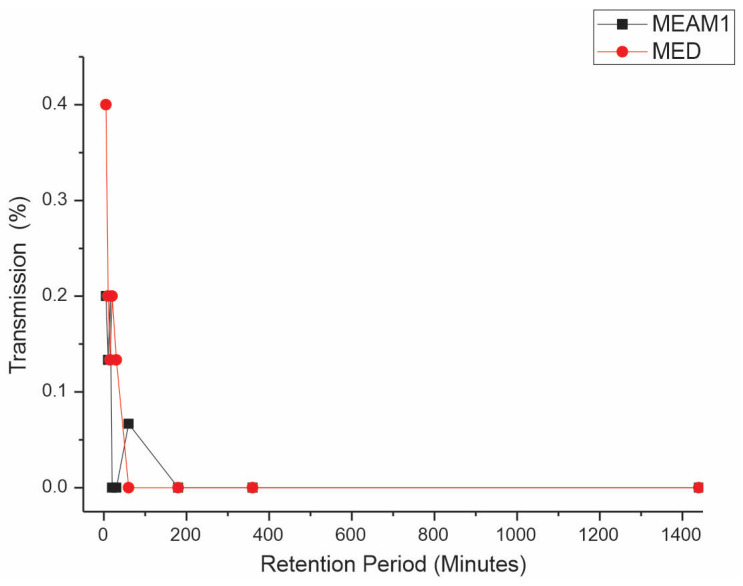
Effect of retention period (RP) on the transmission of cowpea mild mottle virus by *Bemisia tabaci* Middle East–Asia Minor 1 and *Bemisia tabaci* Mediterranean. The differences were estimated by the Mann–Whitney test at a significant level of 0.01 (*n* = 30).

*Retention period (RP).* The Mann–Whitney test reveals significant differences, comparing the retention periods (RPs) between MEAM1 and MED at a significance level of 0.05. The average RPs of CPMMV by the MEAM1 and MED adults are listed in Table 3. The interval that does not include zero suggests a meaningful difference between the two species. The *p*-value for the hypothesis test η1 = η2 versus η1 ≠ η2 is 0.0024, indicating a significant difference. When adjusted for the ties, the *p*-value is also significant at 0.0017. This result demonstrates that MED whiteflies retain the virus for a substantially longer period compared to that of MEAM1 whiteflies. After a 24 h AAP of feeding on CPMMV-infected soybean plants, virus retention by the whiteflies on cotton, a virus non-host plant, was assessed. The transmission efficiency for MED decreased from 40% to 13.3% in the range of 5 min to 30 min, respectively, and no transmission occurred in the range of 1–24 h (Figure 3). For MEAM1, retention periods of 5, 10, and 15 min had an efficiency of 20, 13.3, and 20%, respectively, but from 20 min to 24 h, no transmission occurred.

### 3.2. Whitefly Survey

**Figure 4:**
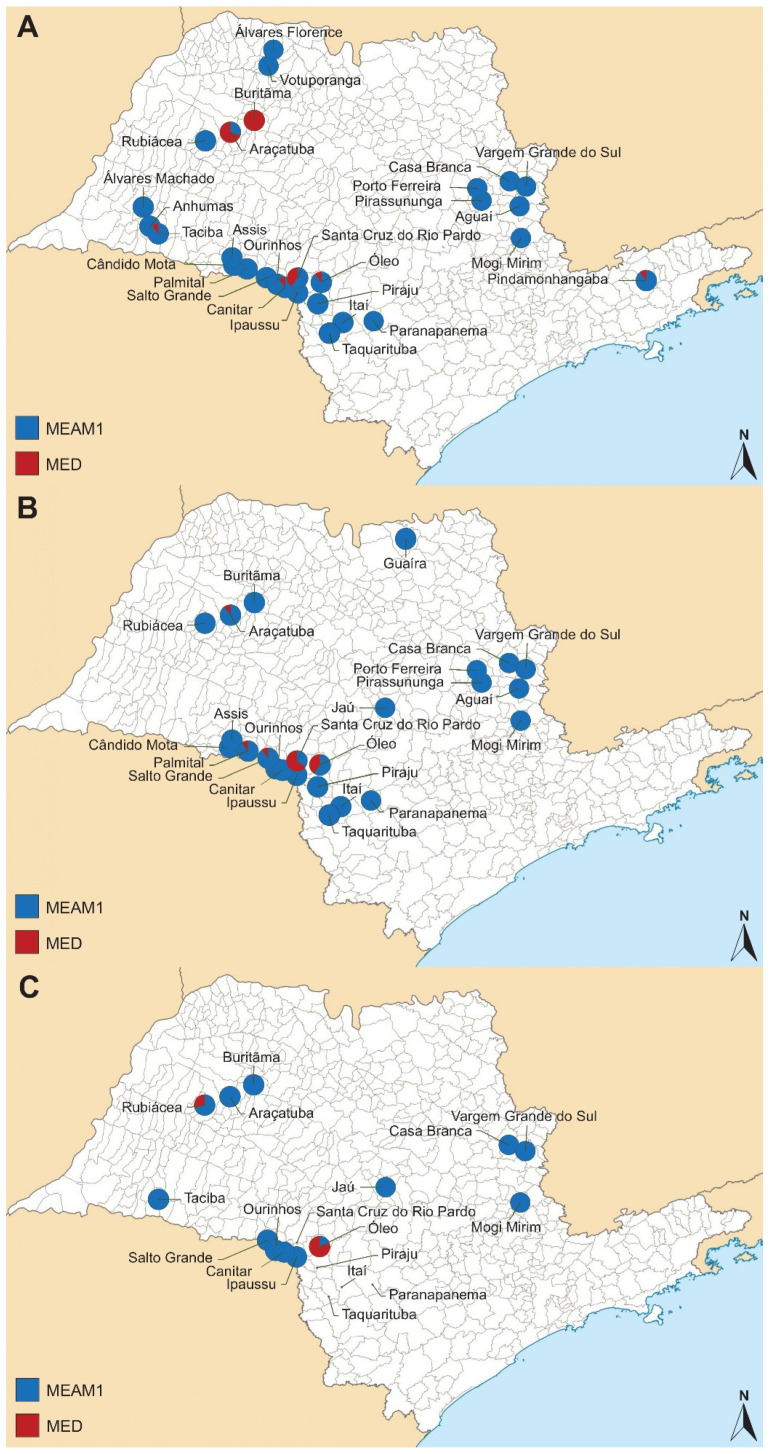
Sampling locations for *Bemisia tabaci* collected in São Paulo State in soybean summer seasons (**A**) 2019/2020, (**B**) 2020/2021, and (**C**) 2021/2022, with species colored in blue (*Bemisia tabaci* Middle East–Asia Minor 1) and red (*Bemisia tabaci* Mediterranean).

A total of 67 collections were conducted over the three soybean seasons: 2019/2020, 2020/2021, and 2021/2022. Details regarding the collection sites, the times of collection, and the geographical coordinates of each sample are summarized in Table 4. In general, a predominance of MEAM1 species was observed across almost all the sampling sites (Table 4, Figure 4). For the first soybean summer season surveyed (2019/2020), 28 sites were sampled, and MED was found in a single infestation in one site (Buritãma), and co-occurred with MEAM1 in six other locations (Araçatuba, Canitar, Óleo, Pindamonhangaba, Santa Cruz do Rio Pardo, and Taciba) (Figure 4A). In the second soybean summer season (2020/2021), MED was found co-occurring with MEAM1 in six out of twenty-four sites collected (Araçatuba, Buritãma, Óleo, Palmital, Salto Grande, and Santa Cruz do Rio Pardo) (Figure 4B). For the last season surveyed (2021/2022), the number of visited sites was reduced to 19 areas, and *B. tabaci* was found in 15 of them. No whiteflies could be collected from four sites because no insects were found infesting soybean (Itaí, Piraju, Santa Cruz do Rio Pardo, and Taquarituba) probably due to the rainy weather. MED was found co-occurring with MEAM1 in three of these sites (Óleo, Paranapenema, and Rubiácea) (Figure 4C).

In some sites, such as Araçatuba, where the incidence of MED was high (70%) in the first season, there was a decrease of 10% in the second season and an absence of MED in the last season. Similarly, the Buritãma site exhibited a comparable dynamic, with the presence of MED at 100%, 20%, and 0 during the first, second, and third seasons, respectively. On the other hand, the Óleo site showed a progressive increase in MED over the three seasons, starting from 10% in the first season, reaching 40% in the second season, and peaking at 80% in the last season. A similar dynamic was observed for Santa Cruz do Rio Pardo, which experienced a progressive increase in MED during the first two seasons (40% and 65%, respectively), but in the last season, there was no presence of *B. tabaci* in this site. In sites such as Canitar, Palmital, Paranapanema, Pindamonhangaba, Rubiácea, Salto Grande, and Taciba, the presence of a small number of MED insects was verified in at least one season.

In the last season, the incidence of *B. tabaci* was very low in several sites, and the insect was even absent in places such as Itaí, Piraju, Santa Cruz do Rio Pardo, and Taquarituba. This was likely related to the weather conditions.

## 4. Discussion

The results of our study indicate the differential transmission dynamics of CPMMV by the *B. tabaci* MEAM1 and MED cryptic species, highlighting MED as a more efficient vector compared to MEAM1. While MEAM1 is still prevalent in the main soybean production areas in São Paulo State, it is possible to observe an increase in MED in open-field conditions in Brazil. These findings raise significant concerns regarding CPMMV management in soybeans [6,7,28].

CPMMV is typically transmitted by whiteflies, and its transmission is considered to occur in a non-persistent manner [8,10,42]. Non-persistent transmission occurs when the vector acquires the virus within seconds or a few minutes and typically retains it for no more than a few minutes to hours [43]. A pioneering study demonstrated that *B. tabaci* MEAM1 was reported to be able to transmit CPMMV from soybean to soybean plants within 10 min of acquisition and inoculation using a group of 15 whiteflies [42]. The recent studies demonstrated that MED species was more efficient at transmitting CPMMV from soybean to soybean plants than MEAM1 based on experiments with groups of 10 insects, with AAPs and IAPs of 24 h [31]. In the present study, we investigated and compared the acquisition, inoculation, and retention of CPMMV in MEAM1 and MED, respectively, in a controlled laboratory setting, employing just one whitefly per plant. Herein, we demonstrated that the transmission efficiency increases with the acquisition period. MED was able to transmit after 10 min of acquisition at an efficiency of 6.6%, reaching its peak transmission efficiency of 100% after an AAP of 24 h. On the other hand, MEAM1 could transmit CPMMV only after an AAP of 6 h, coinciding with its peak of 46.6%, and decreasing to 20% when the acquisition extended to 24 h. Examining the inoculation period, MED was able to transmit CPMMV with an IAP of only 2 min, while MEAM1 required 30 min, achieving efficiencies of 40% and 16.6%, respectively. The highest transmission rate by MED, 93.3%, was observed with an IAP of 6 h. Coincidentally, MEAM1 also reached its peak transmission rate of 46.6% with an IAP of 6 h. The retention of CPMMV persisted in MEAM1 for only 15 min, while it was retained for 30 min in MED. As far as we know, this is the first study to compare the retention periods of CPMMV by MEAM1 and MED, and our data support a semi-persistent type of virus–vector relationship for both the cryptic species. A previous study using 10 whiteflies per plant and only a 24 h AAP also observed that MED was more efficient compared to MEAM1 in transmitting CPMMV from soybean to soybean and from bean to bean [31].

The evidence of the spreading and colonization of MED to soybean plants in Brazil is new [31]. Here, we observed the possible displacement of MEAM1 by MED in some soybean-producing areas of São Paulo State, as verified in Óleo and Santa Cruz do Rio Pardo, where an increase in the MED population was observed over the three years of the survey. This could be related to sweet pepper greenhouses located near the areas of soybean production (personal information). However, a decrease in the MED population during the three years was clearly verified in areas like Araçatuba, Buritãma, and Canitar. It is difficult to predict what will happen with MED insects in the soybean areas in Brazil and whether the insect will adapt well to open-field conditions, considering it is well characterized as an insect that prefers greenhouse environments [44,45,46].

A previous study evaluating the behavior of MED and MEAM1 on soybean in the absence of insecticides showed that MEAM1 and MED do not outcompete each other on this crop and can co-occur in a plant in mixed infestations [47]. However, under conditions of excess insecticide use, MED is highly competitive and causes the displacement of the MEAM1 species [48,49,50,51]. In our studies, we observed that in most areas, MED was found in a mixed infestation with MEAM1, supporting the data obtained by Watanabe et al. (2019) that both can co-exist well on soybean plants. It is also interesting to mention that MED was also found as the predominant species (in single infestation) in areas like Buritãma. Whether this is related to whitefly management and the variety/amount of insecticide use, we cannot affirm, and more studies are necessary to verify the influence of these parameters on the *B. tabaci* dynamic. It is well known that MED species have low susceptibility to insecticides used to control whiteflies [29,50]. Besides the possible increase in the cost of production, the threat of MED to soybean systems can also be related to the possible introduction of new viruses to soybean and other crops, which happened in the early 1990s in Brazil when outbreaks of begomoviruses infecting *Solanaceae* occurred after MEAM1 detection [52].

In conclusion, our results indicate that CPMMV transmission is likely significantly influenced by the *B. tabaci* cryptic species. The rate of transmission is expected to increase with the prevalence of MED over MEAM1 because MED is a more efficient vector. The detection of MED species of *B. tabaci* in soybeans requires the close monitoring of its expansion to other states. Additionally, the capacity of MED to transmit CPMMV and its reduced susceptibility to pesticides are critical concerns that require further investigations and management strategies. Soybean growers should be aware of the potential displacement of MEAM1 by MED, particularly in areas with excess insecticide use, and the associated risk of increased virus transmission. It is crucial to adapt management practices to address the spread of MED and mitigate its impact on soybean crops.

## Figures and Tables

**Table 1:** Different acquisition access periods and efficiencies of transmission of cowpea mild mottle virus by single individuals of *Bemisia tabaci* MEAM1 and MED species.

	Plants Infected/Plants Exposed ^a,b^	
Acquisition Access Period	MEAM1	MED
5 min	0/30 (0)	0/30 (0)
10 min	0/30 (0)	2/30 (6.6)
15 min	0/30 (0)	4/30 (13.3)
20 min	0/30 (0)	16/30 (53.3)
30 min	0/30 (0)	14/30 (46.6)
1 h	0/30 (0)	14/30 (46.6)
3 h	0/30 (0)	24/30 (80.0)
6 h	14/30 (46.6)	28/30 (93.3)
24 h	06/30 (20.0)	30/30 (100.0)

^a^ One viruliferous adult was transferred to each soybean seedling and allowed a 24 h inoculation access period. ^b^ Data are given as *n* (%).

**Table 2:** Different inoculation access periods and efficiencies of the transmission of cowpea mild mottle virus by single individuals of *Bemisia tabaci* MEAM1 and MED species.

	Plants Infected/Plants Exposed ^b,c^	
Inoculation Access Period ^a^	MEAM1	MED
2 min	0/30 (0)	12/30 (40.0)
5 min	0/30 (0)	12/30 (40.0)
10 min	1/30 (3.3)	12/30 (40.0)
20 min	0/30 (0)	16/30 (53.3)
30 min	5/30 (16.6)	18/30 (60.0)
1 h	6/30 (20.0)	18/30 (60.0)
2 h	09/30 (30.0)	16/30 (53.3)
3 h	5/30 (16.6)	22/30 (73.3)
6 h	14/30 (46.6)	28/30 (93.3)
12 h	7/30 (23.3)	20/30 (66.6)
24 h	8/30 (26.6)	20/30 (66.6)

^a^ All insects were given a 24 h acquisition access period. ^b^ One viruliferous adult was transferred to each soybean seedling and allowed a 24 h inoculation access period. ^c^ Data are given as *n* (%).

**Table 3:** Influence of different retention periods on the efficiency of cowpea mild mottle virus transmission by single individuals of *Bemisia tabaci* MEAM1 and MED species.

	Plants Infected/Plants Exposed ^b,c^	
Retention Period ^a^	MEAM1	MED
5 min	6/30 (20.0)	12/30 (40.0)
10 min	4/30 (13.3)	6/30 (20.0)
15 min	6/30 (20.0)	4/30 (13.3)
20 min	0/30 (0)	6/30 (20.0)
30 min	0/30 (0)	4/30 (13.3)
1 h	2/30 (6.6)	0/30 (0)
3 h	0/30 (0)	0/30 (0)
6 h	0/30 (0)	0/30 (0)
24 h	0/30 (0)	0/30 (0)

^a^ All the insects were given a 24 h acquisition access period before undergoing the retention period, and then a 24 h inoculation access period for the soybean seedlings after the retention period. ^b^ One viruliferous adult was transferred to each soybean seedling and allowed a 24 h inoculation period access. ^c^ The data are given as *n* (%).

**Table 4:** The survey of *Bemisia tabaci* in the main soybean growing areas in São Paulo State, Brazil, between the soybean summer seasons 2019/2020, 2020/2021, and 2021/2022.

ID	Collection Site	Coordinates	2019/2020		2020/2021		2021/2022	
			MED ^a^	MEAM1 ^a^	MED ^a^	MEAM1 ^a^	MED ^a^	MEAM1 ^a^
1	Aguaí	22°04′16″ S 46°57′25″ W	0	100	0	100	NC	NC
2	Alvarez Florença	20°17′12″ S 49°55′12″ W	0	100	NC	NC	NC	NC
3	Alvarez Machado	22°01′06″ S 51°24′37″ W	0	100	NC	NC	NC	NC
4	Anhumas	22°27′03″ S 51°26′01″ W	0	100	NC	NC	NC	NC
5	Araçatuba	21°10′33″ S 50°31′33″ W	70	30	10	90	0	100
6	Assis	22°40′48″ S 50°21′05″ W	0	100	0	100	NC	NC
7	Buritãma	21°04′08″ S 50°12′08″ W	100	0	20	80	0	100
8	Cândido Mota	22°44′02″ S 50°21′26″ W	0	100	0	100	NC	NC
9	Canitar	23°00′48″ S 49°46′25″ W	10	90	0	100	0	100
10	Casa Branca	21°55′48″ S 47°04′08″ W	0	100	0	100	0	100
11	Guaíra	20°04′03″ S 48°04′04″ W	NC	NC	0	100	0	100
12	Ipaussu	23°05′11″ S 49°33′03″ W	0	100	0	100	0	100
13	Itaí	23°19′07″ S 49°05′17″ W	0	100	0	100	NP	NP
14	Jaú	22°14′42″ S 48°33′20″ W	NC	NC	0	100	0	10
15	Mogi Mirim	22°28′36″ S 47°03′36″ W	0	100	0	100	0	100
16	Óleo	22°56′33″ S 49°26′14″ W	10	90	40	60	80	20
17	Ourinhos	22°55′57″ S 49°53′13″ W	0	100	0	100	0	100
18	Palmital	22°45′40″ S 50°11′30″ W	0	100	10	90	NC	NC
19	Paranapanema	23°28′26″ S 48°45′16″ W	0	100	0	100	15	85
20	Pindamonhangaba	22°52′05″ S 45°28′18″ W	10	90	NC	NC	NC	NC
21	Piraju	23°16′13″ S 49°16′30″ W	0	100	0	100	NP	NP
22	Pirassununga	22°03′45″ S 47°30′01″ W	0	100	0	100	NC	NC
23	Porto Ferreira	21°49′51″ S 47°27′10″ W	0	100	0	100	NC	NC
24	Rubiácea	21°25′40″ S 50°49′05″ W	0	100	0	100	30	70
25	Salto Grande	22°50′13″ S 50°01′16″ W	0	100	10	90	0	100
26	Sta. Cruz Rio Pardo	22°56′09″ S 49°32′42″ W	40	60	65	35	NP	NP
27	Taciba	22°24′38″ S 51°19′30″ W	10	90	NC	NC	0	100
28	Taquarituba	23°31′27″ S 49°16′15″ W	0	100	0	100	NP	NP
29	Vargem Grande do Sul	21°50′07″ S 46°54′40″ W	0	100	0	100	0	100
30	Votuporanga	20°27′30″ S 50°03′52″ W	0	100	NC	NC	NC	NC
Total of Sites Surveyed			28		24		15	

NC: not collected; NP: not present (areas where whiteflies were not found during the survey); ^a^ Data in percentage.

## Data Availability

The data presented in this study are available on reasonable request from the corresponding author.

## Supplementary Materials

The following supporting information can be downloaded at: https://www.mdpi.com/article/10.3390/insects15080624/s1, Table S1: Primers used for whiteflies and virus identification.

## References

[1] Costa A.S., Gaspar J.O., Vega J. (1983). Mosaico Angular Do Feijão Jalo Causado Por Um Carlavírus Transmitido Pela Mosca Branca *Bemisia tabaci*. Fitopatol. Bras..

[2] Almeida A.M.R., Piuga F.F., Kitajima E.W., Gaspar J.O., Valentin N., Benato L.C., Marin S.R.R., Binneck E., de Oliveira T.G., Belitani P. (2003). Necrose Da Haste Da Soja. Ser. Doc..

[3] Almeida A.M.R. (2008). Viroses Da Soja No Brasil: Sintomas, Etiologia e Controle. Ser. Doc..

[4] Zanardo L., Silva F., Lima A., Milanesi D., Castilho-Urquiza G., Almeida A., Zerbini F., Carvalho C. (2014). Molecular Variability of Cowpea Mild Mottle Virus Infecting Soybean in Brazil. Arch. Virol..

[5] Zanardo L.G., Silva F.N., Bicalho A.A.C., Urquiza G.P.C., Lima A.T.M., Almeida A.M.R., Zerbini F.M., Carvalho C.M. (2014). Molecular and Biological Characterization of *Cowpea Mild Mottle Virus* Isolates Infecting Soybean in Brazil and Evidence of Recombination. Plant Pathol..

[6] Barreto da Silva F., Muller C., Bello V.H., Watanabe L.F.M., Rossitto De Marchi B., Fusco L.M., Ribeiro-Junior M.R., Minozzi G.B., Vivan L.M., Tamai M.A. (2020). Effects of Cowpea Mild Mottle Virus on Soybean Cultivars in Brazil. PeerJ.

[7] Muniyappa V., Reddy D.V.R. (1983). Transmission of Cowpea Mild Mottle Virus by *Bemisia tabaci* in a Nonpersistent Manner. Plant Dis..

[8] Jeyanandarajah P., Brunt A.A. (1993). The Natural Occurrence, Transmission, Properties and Possible Affinities of Cowpea Mild Mottle Virus. J. Phytopathol..

[9] Marubayashi J.M., Yuki V.A., Wutke E.B. (2010). Transmissão Do *Cowpea Mild Mottle Virus* Pela Mosca Branca Bemisia Tabaci Biótipo B Para Plantas de Feijão e Soja. Summa Phytopathol..

[10] Lowe S., Browne M., Boudjelas S., de Poorter M. (2000). 100 of the World’s Worst Invasive Alien Species: A Selection from the Global Invasive Species Database.

[11] de Barro P.J., Liu S.-S., Boykin L.M., Dinsdale A.B. (2011). *Bemisia tabaci*: A Statement of Species Status. Annu. Rev. Entomol..

[12] Boykin L.M., de Barro P.J. (2014). A Practical Guide to Identifying Members of the *Bemisia tabaci* Species Complex: And Other Morphologically Identical Species. Front. Ecol. Evol..

[13] Kanakala S., Ghanim M. (2019). Global Genetic Diversity and Geographical Distribution of *Bemisia tabaci* and Its Bacterial Endosymbionts. PLoS ONE.

[14] Brown J.K., Frohlich D.R., Rosell R.C. (1995). The Sweetpotato or Silverleaf Whiteflies: Biotypes of *Bemisia tabaci* or a Species Complex?. Annu. Rev. Entomol..

[15] Perring T.M. (2001). The Bemisia Tabaci Species Complex. Crop Prot..

[16] Bedford I.D., Briddon R.W., Brown J.K., Rosell R.C., Markham P.G. (1994). Geminivirus Transmission and Biological Characterization of *Bemisia tabaci* (Gennadius) Biotypes from Different Geographic Regions. Ann. Appl. Biol..

[17] Sánchez-Campos S., Navas-Castillo J., Camero R., Soria C., Díaz J.A., Moriones E. (1999). Displacement of Tomato Yellow Leaf Curl Virus (TYLCV)-Sr by TYLCV-Is in Tomato Epidemics in Spain. Phytopathology.

[18] Horowitz A.R., Kontsedalov S., Khasdan V., Ishaaya I. (2005). Biotypes B and Q of Bemisia Tabaci and Their Relevance to Neonicotinoid and Pyriproxyfen Resistance. Arch. Insect Biochem. Physiol..

[19] Bellows T.S., Perring T.M., Gill R.J., Headrick D.H. (1994). Description of a Species of Bemisia (Homoptera: Aleyrodidae). Ann. Entomol. Soc. Am..

[20] Brown J.K., Paredes-Montero J.R., Stocks I.C. (2023). The *Bemisia tabaci* Cryptic (Sibling) Species Group—Imperative for a Taxonomic Reassessment. Curr. Opin. Insect Sci..

[21] Tay W.T., Evans G.A., Boykin L.M., de Barro P.J. (2012). Will the Real *Bemisia tabaci* Please Stand up?. PLoS ONE.

[22] Hu J., de Barro P., Zhao H., Wang J., Nardi F., Liu S.-S. (2011). An Extensive Field Survey Combined with a Phylogenetic Analysis Reveals Rapid and Widespread Invasion of Two Alien Whiteflies in China. PLoS ONE.

[23] Gauthier N., Clouet C., Perrakis A., Kapantaidaki D., Peterschmitt M., Tsagkarakou A. (2014). Genetic Structure of *Bemisia tabaci* Med Populations from Home-Range Countries, Inferred by Nuclear and Cytoplasmic Markers: Impact on the Distribution of the Insecticide Resistance Genes. Pest Manag. Sci..

[24] Lourenção A., Nagai H. (1994). Outbreaks of *Bemisia tabaci* in the São Paulo State, Brazil. Bragantia.

[25] Moraes L.A., Muller C., Bueno R.C.O.D.F., Santos A., Bello V.H., de Marchi B.R., Watanabe L.F.M., Marubayashi J.M., Santos B.R., Yuki V.A. (2018). Distribution and Phylogenetics of Whiteflies and Their Endosymbiont Relationships after the Mediterranean Species Invasion in Brazil. Sci. Rep..

[26] Barbosa L.F., Yuki V.A., Marubayashi J.M., de Marchi B.R., Perini F.L., Pavan M.A., de Barros D.R., Ghanim M., Moriones E., Navas-Castillo J. (2015). First Report of *Bemisia tabaci* Mediterranean (Q Biotype) Species in Brazil. Pest Manag. Sci..

[27] Bello V.H., Watanabe L.F.M., Fusco L.M., de Marchi B.R., Barreto da Silva F., Gorayeb E.S., Moura M.F., de Souza I.M., Muller C., Salas F.J.S. (2020). Outbreaks of *Bemisia tabaci* Mediterranean Species in Vegetable Crops in São Paulo and Paraná States, Brazil. Bull. Entomol. Res..

[28] Krause-Sakate R., Watanabe L.F.M., Gorayeb E.S., da Silva F.B., Alvarez D.D.L., Bello V.H., Nogueira A.M., de Marchi B.R., Vicentin E., Ribeiro-Junior M.R. (2020). Population Dynamics of Whiteflies and Associated Viruses in South America: Research Progress and Perspectives. Insects.

[29] Moraes L.A., Marubayashi J.M., Yuki V.A., Ghanim M., Bello V.H., de Marchi B.R., da Fonseca Barbosa L., Boykin L.M., Krause-Sakate R., Pavan M.A. (2017). New Invasion of *Bemisia tabaci* Mediterranean Species in Brazil Associated to Ornamental Plants. Phytoparasitica.

[30] Bello V.H., Barreto da Silva F., Watanabe L.F.M., Vicentin E., Muller C., de Freitas Bueno R.C.O., Santos J.C., De Marchi B.R., Nogueira A.M., Yuki V.A. (2021). Detection of *Bemisia tabaci* Mediterranean Cryptic Species on Soybean in São Paulo and Paraná States (Brazil) and Interaction of Cowpea Mild Mottle Virus with Whiteflies. Plant Pathol..

[31] Pan H., Preisser E.L., Chu D., Wang S., Wu Q., Carrière Y., Zhou X., Zhang Y. (2015). Insecticides Promote Viral Outbreaks by Altering Herbivore Competition. Ecol. Appl..

[32] Wang W., Wang S., Han G., Du Y., Wang J. (2017). Lack of Cross-Resistance between Neonicotinoids and Sulfoxaflor in Field Strains of Q-Biotype of Whitefly, *Bemisia tabaci*, from Eastern China. Pestic. Biochem. Physiol..

[33] Valdes C., Gillespie J., Dohlman E.N. Soybean Production, Marketing Costs, and Export Competitiveness in Brazil and the United States. 2023. (Report No. EIB-262). U.S. Department of Agriculture, Economic Research Service. https://search.nal.usda.gov/discovery/fulldisplay?context=L&vid=01NAL_INST:MAIN&docid=alma9916417433407426.

[34] United States Department of Agriculture Foreign Agricultural Service (2024). Oilseeds and Products Update. https://fas.usda.gov/data/brazil-oilseeds-and-products-update-36.

[35] Martins V.A., Fredo C.E., Baptistella C.S.L., Ghobril C.N., Bini D.L.C., Camargo F.P., Angelo J.A., Miura M., Coelho P.J., Nakama L.M. (2023). Previsões e Estimativas das Safras Agrícolas do Estado de São Paulo, Ano Agrícola 2022/23, Fevereiro de 2023. Anál. Indicadores Agronegócio.

[36] Vieira S.S., Bueno R.C.O.D.F., Bueno A.D.F., Boff M.I.C., Gobbi A.L. (2013). Different Timing of Whitefly Control and Soybean Yield. Ciênc. Rural.

[37] Navas-Castillo J., Fiallo-Olivé E., Sánchez-Campos S. (2011). Emerging Virus Diseases Transmitted by Whiteflies. Annu. Rev. Phytopathol..

[38] Walsh P.S., Metzger D.A., Higuchi R. (1991). Chelex-100 as a Medium for Simple Extraction of DNA for PCR-Based Typing from Forensic Material. Biotechniques.

[39] de Barro P.J., Scott K.D., Graham G.C., Lange C.L., Schutze M.K. (2003). Isolation and Characterization of Microsatellite Loci in *Bemisia tabaci*. Mol. Ecol. Notes.

[40] Skaljac M., Zanic K., Ban S.G., Kontsedalov S., Ghanim M. (2010). Co-Infection and Localization of Secondary Symbionts in Two Whitefly Species. BMC Microbiol..

[41] R Core Team (2019). R: A Language and Environment for Statistical Computing.

[42] Iwaki M. (1982). Whitefly Transmission and Some Properties of Cowpea Mild Mottle Virus on Soybean in Thailand. Plant Dis..

[43] Ng J.C.K., Falk B.W. (2006). Virus-Vector Interactions Mediating Nonpersistent and Semipersistent Transmission of Plant Viruses. Annu. Rev. Phytopathol..

[44] Kontsedalov S., Abu-moch F., Lebedev G., Czosnek H., Horowitz A.R. (2012). *Bemisia tabaci* Biotype Dynamics and Resistance to Insecticides in Israel during the Years 2008–2010. J. Integr. Agric..

[45] McKenzie C.L., Bethke J.A., Byrne F.J., Chamberlin J.R., Dennehy T.J., Dickey A.M., Gilrein D., Hall P.M., Ludwig S., Oetting R.D. (2012). Distribution of *Bemisia tabaci* (Hemiptera: Aleyrodidae) Biotypes in North America after the Q Invasion. J. Econ. Entomol..

[46] Parrella G., Scassillo L., Giorgini M. (2012). Evidence for a New Genetic Variant in the *Bemisia tabaci* Species Complex and the Prevalence of the Biotype Q in Southern Italy. J. Pest Sci..

[47] Watanabe L.F.M., Bello V.H., de Marchi B.R., Barreto da Silva F., Fusco L.M., Sartori M.M.P., Pavan M.A., Krause-Sakate R. (2019). Performance and Competitive Displacement of *Bemisia tabaci* MEAM1 and MED Cryptic Species on Different Host Plants. Crop Prot..

[48] Yao F.L., Zheng Y., Huang X.Y., Ding X.L., Zhao J.W., Desneux N., He Y.X., Weng Q.Y. (2017). Dynamics of *Bemisia tabaci* Biotypes and Insecticide Resistance in Fujian Province in China during 2005–2014. Sci. Rep..

[49] Nauen R., Denholm I. (2005). Resistance of Insect Pests to Neonicotinoid Insecticides: Current Status and Future Prospects. Arch. Insect Biochem. Physiol..

[50] Horowitz A.R., Ishaaya I. (2014). Dynamics of Biotypes B and Q of the Whitefly *Bemisia tabaci* and Its Impact on Insecticide Resistance. Pest Manag. Sci..

[51] Gerling D., Motro U., Horowitz R. (1980). Dynamics of *Bemisia tabaci* (Gennadius) (Homoptera: Aleyrodidae) Attacking Cotton in the Coastal Plain of Israel. Bull. Entomol. Res..

[52] Faria J.C., Bezerra I.C., Zerbini F.M., Ribeiro S.G., Lima M.F. (2000). Situação Atual Das Geminiviroses No Brasil. Fitopatol. Bras..

