# Supplementary material for: Exploring Bemisia tabaci Middle East–Asia Minor I and Mediterranean Cryptic Species Relationship with Cowpea Mild Mottle Virus and Their Dynamics in Soybean Fields
Source: Insects. 2024 Aug 19;15(8):624. doi: 10.3390/insects15080624 (PMC11354603; doi:10.3390/insects15080624)
Supplement: Supplementary file 1 [file insects-15-00624-s001.zip › insects-3108612-supplementary.pdf]

Table S1. Primers used for whiteflies and virus identification.

Identification of whiteflies and virus	Target gene	Primer sequence (5’>3’)	Tm (°C)	Reference
MEAM1/MED differentiation	microsatellite locus	Bem23F- CGGAGCTTGCGCCTTAGTC Bem23R-CGGCTTTATCATAGCTCTCGT	55	37
CPMMV	Coat protein region	1280F- GGCGTTCCAAAAGCTGCCGAT 1696R-GGAGCCACCTTTCCAATCAA	55	36
